# A longitudinal study of alcohol consumption among adults in Victoria, Australia during the COVID-19 pandemic

**DOI:** 10.1371/journal.pone.0313599

**Published:** 2024-12-09

**Authors:** Tianhui Ke, Michael Livingston, Yanqin Zhang, Damian Pavlyshyn, Aimée Altermatt, Alexander Thomas, Thi Nguyen, Shelley Walker, Sophie Hill, Alison Coelho, Alisa Pedrana, Mark Stoové, Margaret Hellard, Katherine B. Gibney, Anna L. Wilkinson

**Affiliations:** 1 Disease Elimination, Burnet Institute, Melbourne, VIC, Australia; 2 Centre for Alcohol Policy Research, La Trobe University, Bundoora, VIC, Australia; 3 National Drug Research Institute, Faculty of Health Sciences, Curtin University, Perth, WA, Australia; 4 School of Public Health and Preventive Medicine, Monash University, Melbourne, VIC, Australia; 5 Centre for Health Communication and Participation, La Trobe University, Bundoora, VIC, Australia; 6 Coelho Networks, Melbourne, VIC, Australia; 7 Melbourne School of Population and Global Health, University of Melbourne, Melbourne, VIC, Australia; 8 Department of Infectious Diseases, The Alfred and Monash University, Melbourne, VIC, Australia; 9 Department of Infectious Diseases, University of Melbourne, at the Peter Doherty Institute for Infection and Immunity, Parkville, VIC, Australia; University of Connecticut Health Center: UConn Health, UNITED STATES OF AMERICA

## Abstract

**Objectives:**

Whilst public health measures were effective in reducing COVID-19 transmission, unintended negative consequences may have occurred. This study aims to assess changes alcohol consumption and the heavy episodic drinking (HED) during the pandemic.

**Methods:**

Data were from the Optimise Study, a longitudinal cohort of Australian adults September 2020–August 2022 that over-sampled priority populations at higher risk of contracting COVID-19, developing severe COVID-19 or experiencing adverse consequences of lockdowns. Frequency of alcohol consumption (mean number of days per week) and past-week HED were self-reported. Generalised linear models estimated the association between time and (1) the frequency of alcohol consumption and (2) heavy episodic drinking.

**Results:**

Data from 688 participants (mean age: 44.7 years, SD:17.0; 72.7% female) and 10,957 surveys were included. Mean days of alcohol consumption per week decreased from 1.92 (SD: 1.92) in 2020 to 1.54 (SD:1.94) in 2022. The proportion of participants reporting HED decreased from 25.4% in 2020 to 13.1% in 2022. During two lockdown periods, known as “lockdown five”, (OR:0.65, 95%CI [0.47,0.90]) and “lockdown six” (OR:0.76, 95%CI [0.67,0.87]), participants were less likely to report HED.

**Conclusions:**

Participants alcohol drinking frequency and HED decreased during the pandemic. This study provides a strong description of alcohol consumption during the pandemic and suggests that lockdowns did not have the unintended consequences of increased alcohol consumption.

## Background

The COVID-19 pandemic has dramatically changed people’s lives since the first case was reported in November 2019. To reduce COVID-19 transmission, many countries implemented public health measures [1]. In the jurisdiction of Victoria, the second most populous state in Australia, the first high-level lockdown was imposed on 30 March 2020. Restrictions involved stay-at-home orders, closures of schools and non-essential businesses, and restrictions on travel [2]. Between 2020 and 2021, the state of Victoria went through six lockdowns, collectively spanning a total of 262 days [3]. Lockdown measures in Victoria were strict and heavily policed, with only five reasons permitted to leave your home (food and supplies, exercise, caregiving, work if necessary and to get vaccinated). During these periods on-premise licensed venues were closed, but takeaway alcohol sales continued [4]. Although these measures were effective in limiting COVID-19 cases, hospitalisations and deaths in Victoria [5], they also restricted access to in-person social interactions, mental health services and physical health facilities, which are strategies many people use during times of increased distress [6]. Therefore, the COVID-19 pandemic created an environment that had the potential to lead to detrimental strategies, such as harmful alcohol consumption [7]. Previous studies indicated that exposure to such community-wide disasters (including natural disasters, pandemics, and terrorism) were associated with increased alcohol use [8–10].

The key mechanisms by which the pandemic may have increased alcohol consumption is via increases in stress. Victorians were subject to a range of stressors including direct concerns about a contagious and deadly disease, increased pressures related to the social isolation linked to the public health restrictions that were implemented and the potential economic impacts of the pandemic in terms of income and employment. A range of studies have highlighted that general stress can increase drinking and alcohol use disorders (e.g., see [11] for a review) and that drinking to cope is a key risk factor for problematic outcomes [12]. Similarly, while population-level studies often show that overall drinking declines during economic downturns (e.g. [13]), individuals who experience unemployment are at increased risk of alcohol use (e.g., [14]).

To date research on the impact of COVID-19 on alcohol consumption has generated mixed evidence. An early systematic review of the global literature that included 45 studies from December 2019 to November 2020 reported a trend towards increased alcohol consumption in the general population [15], while a cross-national European study found an average decrease in alcohol consumption and a reduced frequency of heavy episodic drinking during the first months of the COVID-19 pandemic [16]. More recent reviews point to significant heterogeneity in COVID-19 impacts between countries, meaning local evidence is key [17]. Australian data has generally showed relatively small impacts of the pandemic. For example, in one of the rare representative surveys conducted in the early part of the pandemic, Biddle et al. [18] found that one in five people reported increased alcohol use in May 2020 since the commencement of the pandemic, while a similar proportion of people reported that they reduced their alcohol consumption. More recent Australian studies have generally shown small declines in consumption [19, 20]. While early studies relied on convenience samples and retrospective measures, more recent survey and wastewater analyses have largely reproduced these findings [21, 22].

Existing studies suggest that some population groups were more likely to report increased alcohol consumption during the COVID-19 pandemic than others. A US national study comparing alcohol consumption in mid-2019 and mid-2020 found increased alcohol use among women, younger and non-Hispanic White participants [23]. A meta-analysis study investigating the factors associated with a change in alcohol consumption during the COVID-19 pandemic found that working remotely and losing income were predictors of increased alcohol consumption [24]. Analysis among samples of Australians pointed to bigger declines in drinking for young people [20] and baseline risky drinkers [19] than for older people and moderate drinkers.

Most Australian research that assessed alcohol consumption during the COVID-19 pandemic is based on cross-sectional, retrospective data [20, 25, 26], and therefore cannot explore changes in alcohol consumption over time. Existing Australian longitudinal studies have only examined the alcohol consumption at the early stage of lockdown or in the first year of the COVID-19 pandemic [7, 27, 28]. Measuring alcohol consumption over time in the pandemic, in a highly characterised group, is important to understand if changes occurred, and in what groups, to inform future pandemic planning. We aim to address this gap in evidence and use data from the Optimise Study, a longitudinal cohort study conducted from September 2020 to August 2022. We examined: (i) changes in the frequency of alcohol consumption and the proportion of heavy episodic drinking over time during the COVID-19 pandemic; (ii) the association between the frequency of alcohol consumption and social demographic characteristics, Christmas and lockdown periods; and (iii) the association between the heavy episodic drinking and social demographic characteristics, Christmas and lockdown periods.

## Methods

### Study design

The Optimise Study was a longitudinal cohort study of adults residing in Victoria, Australia.

### Setting

The study was established to examine how the community managed and responded to the COVID-19 pandemic, including measuring the impact of public health restrictions aimed at reducing COVID-19 transmission.

### Participants

Participants were recruited from 27 September 2020 to 18 December 2021, and were regularly followed up until August 2022. Optimise was not designed to be a representative sample but an in-depth study of priority populations. Thus, participants were intentionally oversampled from key groups considered at risk of: (i) contracting COVID-19; (ii) developing severe COVID-19; or (iii) experiencing adverse consequences of the restrictions. Eligible participants were aged 18 years or older, resided in Victoria, Australia, and provided verbal informed consent with research staff, recorded in the study database, due to public health restrictions at the time of recruitment precluding face-to-face contact with participants. Participants were recruited through paid and unpaid social media advertisements and flyers promoted through community and industry groups, community-based organisations, and social and professional networks. More detailed description of Optimise participants and methods can be found elsewhere [29–31].

### Data measurement

Optimise participants were asked to complete a baseline survey and then monthly follow-up surveys, either self-completed online or in an interview with research staff. Participants included in the present study completed a baseline survey and at least two follow-up surveys, and completed their surveys in English.

We include data on key socio-demographic and pandemic-related measures in our analyses: participants’ age, gender (woman/man/other), education (high school or lower/TAFE or trade certificate/undergraduate/postgraduate), employment status (full time or self-employed/part time or casual/not employed/retired), working environment (attend workplace/work from home/both workplace and working from home/not working), place of residence (metropolitan/region), chronic health condition (yes/no), and high-risk worker status (yes/no). In the present study, we grouped aged care, hotel quarantine or COVID-19 border control, and healthcare workers into a broad ‘high risk worker’ category.

The primary outcome was the frequency of alcohol consumption (count of days), measured using the self-reported number of days in the past week that a participant consumed alcohol. Frequency of alcohol consumption was measured in both the baseline survey and each month’s follow-up survey using the question ‘In the past week, please estimate how many days per week did you consume any alcohol?’, with options of 0–7 days.

The secondary outcome was heavy episodic drinking in the past week (yes/no). Heavy episodic drinking was measured by the question ‘In the past week, please estimate how many days per week did you consume six or more alcoholic drinks in a single day’ in baseline and follow-up surveys, asked of all participants who indicated consuming alcohol at least one day in the past week. Participants were presented options of 0–7 days. A binary variable (yes/no) was then created for the analysis purposes, with ‘0 days’ = no and those who answered ‘0 days’ to the consumption question described above also coded as no, and ‘1 day’ to ‘7 days’ = yes.

The study period of October 2020–August 2022 included five lockdown periods in Victoria. To estimate the association between alcohol consumption and lockdown periods, we derived a categorical variable (non-lockdown period/lockdown 2/lockdown 4/lockdown 5/lockdown 6) according to participants’ survey completion date. Lockdown 3 (13–17^th^ Feb 2021) was classified as a non-lockdown period in the analysis due to the short period of time (5 days).

Due to prior evidence of increases in alcohol consumption during the Christmas and New Year period [32, 33], to account for confounding, we derived a categorical variable according to participants’ survey completion date, with three categories (non-Christmas period/Christmas 2020/Christmas 2021). We classified the last three weeks of December and the first week of January as the Christmas period (e.g., 14^th^ December 2020 – 10^th^ January 2021 as Christmas 2020).

### Statistical methods

Descriptive analysis summarised the demographic characteristics of the sample. To summarise the frequency of alcohol consumption, we calculated the mean and standard deviation (SD) of the number of days of alcohol consumption per week separately for socio-demographic groups and calendar years. To summarise heavy episodic drinking, the proportion of participants’ heavy drinking was reported by different socio-demographic groups and calendar years.

To estimate the associations between frequency of alcohol consumption in the past week (number of days), time and all the covariates, we fitted a generalised linear model for count data. The covariates included time, socio-demographic characteristics, Christmas period and lockdown period. Time (in one-week blocks) was a continuous variable from 1 to 101, representing the first week of October 2020 to the last week of August 2022. Exploratory analysis confirmed that the data had excess zeroes (41.5% of surveys had zero-days of alcohol drinking) and therefore a Zero-inflated Poisson model was fitted. Participants with complete data on all covariates were included in the model. The model parameters are presented as rate ratios (RR) and 95% CIs. The inherent correlation in the data due to the inclusion of multiple surveys per person was accounted for by using bootstrapped confidence intervals (CIs). Previous literature suggests that for Zero-inflated Poisson models, bootstrapping provides reliable confidence intervals compared to other methods [34]. Therefore, 95% CIs for model coefficients were calculated using a non-parametric bootstrap with 200 samples.

A generalised linear model was used to estimate the association between the binary outcome of heavy episodic drinking and time, socio-demographic characteristics, and Christmas and lockdown periods, accounting for repeated measures with generalised estimating equations (GEEs). GEEs offer the benefit of minimal assumptions about missingness and tolerate misspecification of the correlation in the data. The model results were reported as odds ratios (ORs), and 95% CIs.

Participants with missing data on covariates were included in descriptive analysis. Only participants with complete data on the outcome and all covariates were included in the regression models (i.e., a complete case analysis).

### Ethical statement

Ethics approval for Optimise was provided by the Alfred Human Research Ethics Committee, Approval Number 333/20. Participants provided verbal informed consent with research staff, recorded in the study database, due to public health restrictions at the time of recruitment precluding face-to-face contact with participants.

All analyses were conducted using R version 4.1.3.

## Results

**Fig 1:**
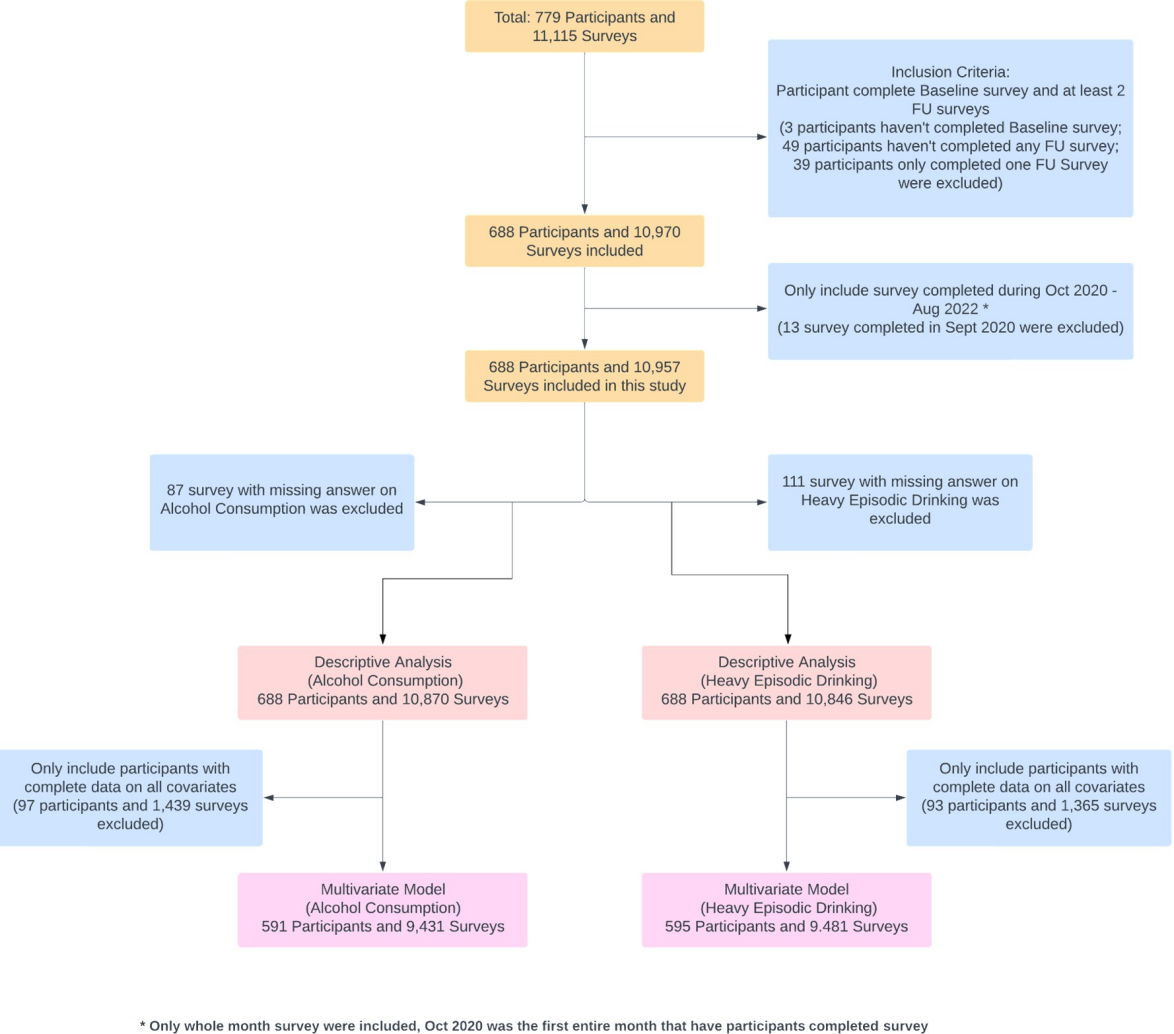
Participant flow chart.

**Fig 2:**
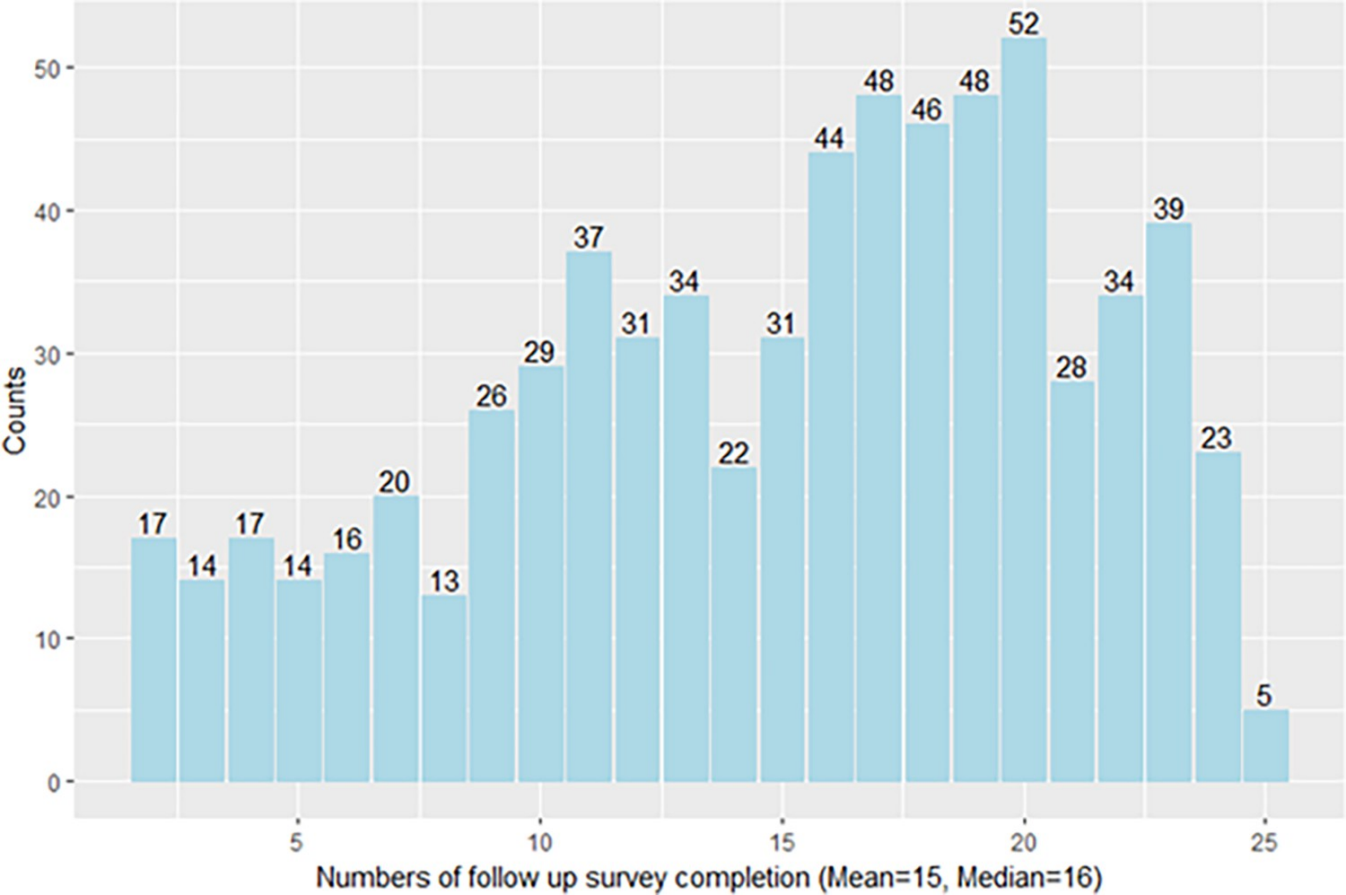
Counts of follow-up surveys completed by Optimise participants, Victoria, Australia, N = 688 participants.

**Fig 3:**
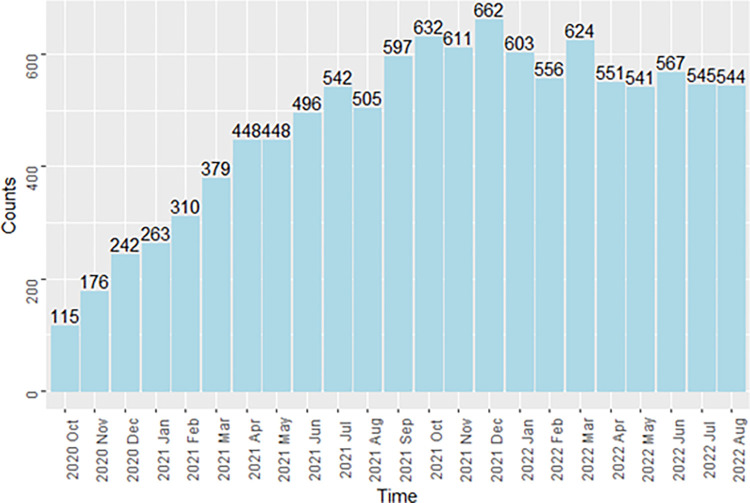
Counts of unique participants who completed a survey each month during October 2020 to August 2022, Optimise, Victoria, Australia, N = 10,957 surveys.

A participant flow chart is shown in Fig 1. In total, 779 participants were recruited into Optimise, of whom 688 (87.3%) completed the baseline survey and at least two follow-up surveys and were included in this study. Of the 688 participants, the mean number of completed follow-up surveys was 15, and the median number was 16 (Fig 2). Because Optimise had rolling recruitment, the number of follow-up surveys completed each month increased until reaching a peak in December 2021. Between July 2021 and August 2022, the total number of participants completing a follow-up survey was consistently above 500. Notably, the number of monthly completed surveys was still high (544) in the last month of the study period (Fig 3).

**Fig 4:**
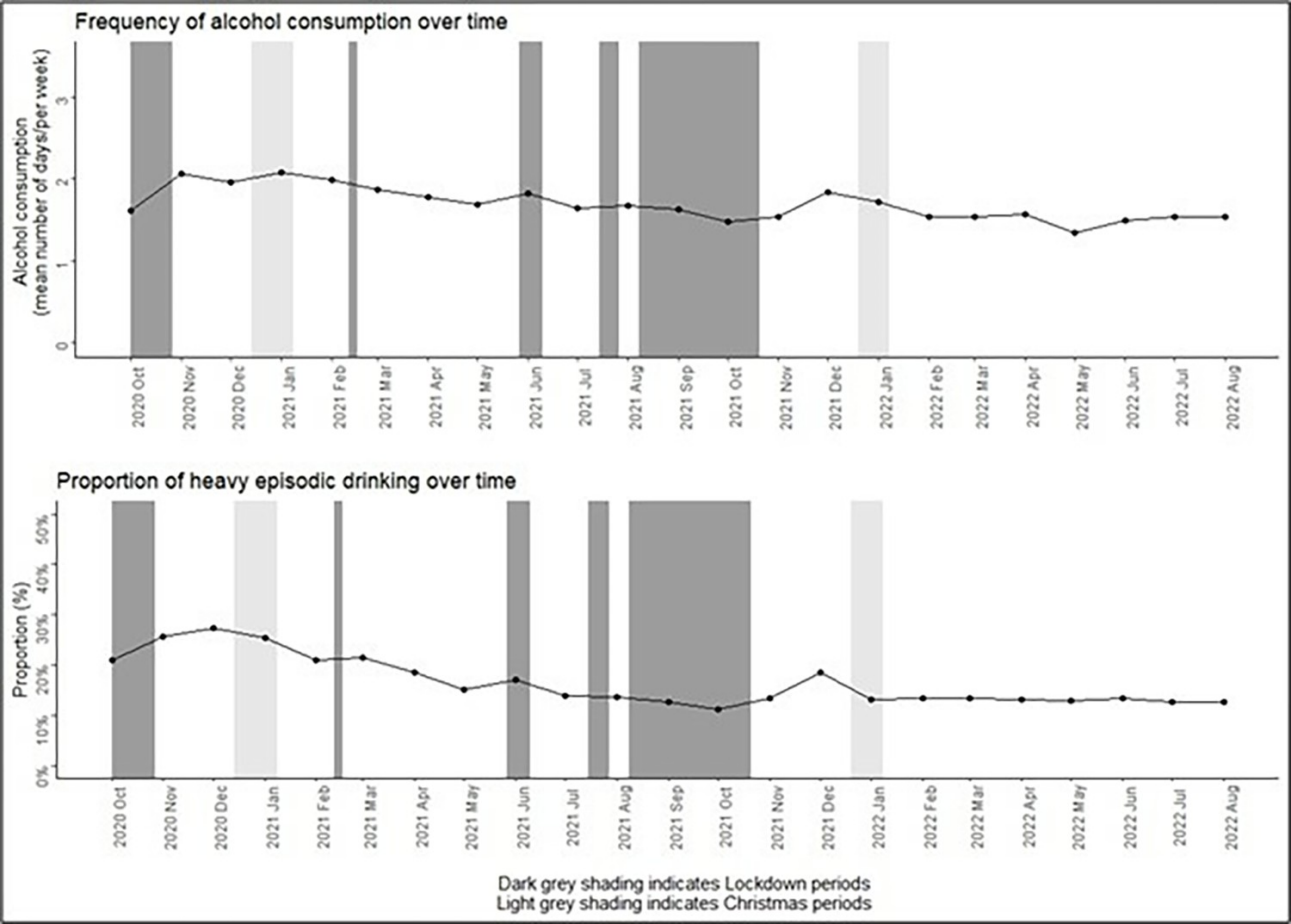
Frequency of alcohol consumption and proportion of heavy episodic drinking by month, October 2020 to August 2022, Optimise, Victoria, Australia, N = 688. * Lockdown 2: 9^th^ July 2020 – 27^th^ October 2020, Lockdown 3: 13^th^ Feb 2021 – 17^th^ Feb 2021, Lockdown 4: 28^th^ May 2021 – 10^th^ June 2021, Lockdown 5: 16^th^ July 2021 – 27^th^ July 2021, Lockdown 6: 5^th^ August 2021 – 21^st^ October 2021.

Among the 688 eligible participants, the mean age at baseline was 44.7 years (SD = 17.0), 72.7% were women, 81.0% lived in metropolitan Melbourne, 28.2% reported living with chronic health conditions, and 19.8% were identified as high-risk workers (Table 1). Across the entire study period of October 2020–August 2022, the mean days of alcohol consumption per week decreased from 1.92 (SD: 1.92) in 2020 to 1.54 (SD: 1.94) in 2022 (Table 2). Mean days of alcohol consumption per week increased slightly during the second lockdown period (October 2020) and 2020 Christmas period with some fluctuations throughout the study period (Fig 4).

**Table 1:** Description of the sociodemographic characteristics of Optimise participants, Victoria, Australia, N = 688.

Demographic	Number	% total
**Age (Years)**	Mean = 44.7	SD = 17.0
Missing	3	
**Age group, years**		
18–24	113	16.4%
25–34	141	20.5%
35–44	104	15.1%
45–54	112	16.3%
55–64	114	16.6%
65+	101	14.7%
Missing	3	0.4%
**Gender**		
Man	181	26.3%
Woman	500	72.7%
Other	4	0.5%
Missing	3	0.4%
**Education**		
High school or lower	118	17.2%
Tertiary education–TAFE/trade certificate	111	16.1%
Tertiary education—undergraduate	257	37.4%
Tertiary education—postgraduate	198	28.8%
Missing	4	0.6%
**Employment status**		
Full-time/Self-employed	243	35.3%
Part-time/Casual	229	33.3%
Not employed	86	12.5%
Retired	105	15.3%
Missing	25	3.7%
**Working environment**		
Attend workplace	224	32.6%
Work from home	132	19.2%
Both workplace and working from home	74	10.8%
Not working	191	27.8%
Missing	67	9.7%
**Place of residence**		
Metro	557	81.0%
Region	124	18.0%
Missing	7	1.0%
**Chronic condition**		
Yes	194	28.2%
No	484	70.4%
Missing	10	1.5%
**High-risk workers** *		
Yes	136	19.8%
No	551	80.1%
Missing	1	0.1%

* High-risk workers: including aged care workers, hotel quarantine or COVID-19 border control workers, and healthcare workers

**Table 2:** Mean number of days of alcohol consumption past week and percentage of heavy episodic drinking of Optimise participants, Victoria, Australia, N = 688.

Demographic	Alcohol consumption		Heavy episodic drinking	
	Mean (SD)	Number of surveys*	Yes (%)	Number of surveys*
**Total**	1.65 (1.95) Median = 1.00	10,870	1,659 (15.2%)	10,846
**Year of survey completion**				
2020	1.92 (1.92)	531	135 (25.4%)	532
2021	1.72 (1.95)	5,836	931 (16.0%)	5,820
2022	1.54 (1.94)	4,503	588 (13.1%)	4,494
**Age group, years**				
18–24	1.21 (1.48)	1,598	381 (23.8%)	1,600
25–34	1.28 (1.54)	2,130	397 (18.7%)	2,128
35–44	1.58 (2.00)	1,558	265 (17.0%)	1,556
45–54	1.86 (2.05)	1,717	304 (17.7%)	1,714
55–64	1.81 (1.88)	2,059	140 (6.8%)	2,051
65+	2.19 (2.45)	1,775	167 (9.5%)	1,764
**Gender**				
Man	1.95 (2.01)	2,711	605 (22.3%)	2,709
Woman	1.55 (1.92)	8,032	1,034 (12.9%)	8,010
**Education**				
High school or lower	1.51 (1.91)	1,768	322 (18.2%)	1,765
Tertiary education–TAFE/trade certificate	1.44 (1.90)	1,834	312 (17.0%)	1,831
Tertiary education—undergraduate	1.67 (1.95)	4,137	666 (16.1%)	4,132
Tertiary education—postgraduate	1.81 (1.97)	3,071	345 (11.3%)	3,059
**Employment status**				
Full-time/Self-employed	1.83 (1.99)	3,791	751 (19.9%)	3,782
Part-time/Casual	1.51 (1.77)	3,499	525 (15.0%)	3,499
Not employed	1.21 (1.66)	1,317	231 (17.6%)	1,315
Retired	1.96 (2.29)	1,880	113 (6.1%)	1,867
**Working environment**				
Attend workplace	1.52 (1.80)	3,315	531 (16.0%)	3,310
Work from home	1.86 (1.96)	2,168	401 (18.5%)	2,163
Both workplace and working from home	2.06 (1.99)	1,205	254 (21.1%)	1,205
Not working	1.65 (2.08)	3,197	344 (10.8%)	3,182
**Place of residence**				
Metro	1.60 (1.92)	8,653	1,333 (15.4%)	8,634
Region	1.87 (2.06)	2,138	307 (14.4%)	2,133
**Chronic condition**				
Yes	1.73 (2.15)	3,211	300 (9.4%)	3,202
No	1.63 (1.86)	7,519	1,343 (17.9%)	7,506
**High-risk workers** **				
Yes	1.49 (1.84)	2,133	1,417 (16.3%)	8,700
No	1.70 (1.97)	8,718	235 (11.0%)	2,127

*Number of surveys completed during the study period

**High-risk workers: including aged care workers, hotel quarantine or COVID-19 border control workers, and healthcare workers

All demographic variables (including Age, Gender, Education, Employment status, Working environment, Place of residence, Chronic condition and high-risk workers) were measured at baseline.

Among the 688 participants, the prevalence of heavy episodic drinking decreased from 25.4% in 2020 to 13.1% in 2022. In contrast to the frequency of alcohol consumption, participants aged 18–25 years (23.8%) reported a higher prevalence of heavy episodic drinking compared with other age groups. Higher prevalence of heavy episodic drinking was also reported among participants who were male (22.3%) compared with female (12.9%); those who had a hybrid working environment (21.1%); or were high-risk workers (16.3%) (Table 2).

Of the 688 eligible participants, 591 participants had complete data on the outcome and all covariates and contributed 9,431 surveys to the regression modelling of frequency of alcohol consumption. Compared to participants aged 18–24 years, those aged 35–44 (adjusted rate ratio (aRR): 1.50; 95% CI: 1.17–1.99), 45–54 (aRR: 1.68; 95% CI: 1.36–2.11), 55–64 (aRR:1.56; 95% CI: 1.24–1.96), and over 65 years (aRR:2.19; 95% CI: 1.69–2.79) reported higher frequency of alcohol consumption during the study period. The frequency of alcohol consumption was associated with Christmas periods compared to the non-Christmas period, the mean number of days per week that participants consumed alcohol was 1.09 times higher (95% CI: 1.01–1.19) during the 2020 Christmas period, and 1.21 times higher (95% CI: 1.11–1.31) during the 2021 Christmas period (Table 3). We did not find evidence of an association between the frequency of alcohol consumption and lockdown periods.

**Table 3:** Association between alcohol consumption frequency and sociodemographic variables, lockdown period and Christmas period. Results of zero-inflated Poisson regression, Optimise, Victoria, Australia, N = 9,431 surveys from 591 participants.

Variable	Alcohol consumption	
	Unadjusted Rate Ratio (95% CI†)	Adjusted Rate Ratio* (95% CI)
**Age group, years**		
18–24	Reference group	Reference group
25–34	1.08 (0.87, 1.34)	1.06 (0.81, 1.31)
35–44	1.43 (1.07, 1.83)	**1.50 (1.17, 1.99)**
45–54	1.60 (1.30, 1.96)	**1.68 (1.36, 2.11)**
55–64	1.45 (1.17, 1.78)	**1.56 (1.24, 1.96)**
65+	1.97 (1.56, 2.44)	**2.19 (1.69, 2.79)**
**Gender**		
Woman	Reference group	Reference group
Man	1.11 (0.95, 1.24)	1.11 (0.96, 1.26)
**Education**		
High school or lower	Reference group	Reference group
Tertiary education–TAFE/trade certificate	1.09 (0.82, 1.37)	0.94 (0.69, 1.20)
Tertiary education—undergraduate	1.07 (0.86, 1.33)	1.10 (0.91, 1.37)
Tertiary education—postgraduate	1.14 (0.90, 1.45)	1.01 (0.81, 1.23)
**Working environment**		
Not working	Reference group	Reference group
Attend workplace	0.83 (0.68, 1.00)	1.01 (0.82, 1.25)
Work from home	0.95 (0.81, 1.13)	1.11 (0.91, 1.34)
Both workplace and working from home	1.01 (0.82, 1.23)	1.24(0.98, 1.54)
**Place of residence**		
Region	Reference group	Reference group
Metro	0.91 (0.77, 1.10)	1.03 (0.84, 1.18)
**Chronic condition**		
No	Reference group	Reference group
Yes	1.21 (1.05, 1.36)	1.04 (0.86, 1.17)
**High-risk workers** **		
No	Reference group	Reference group
Yes	0.98 (0.82, 1.14)	1.00 (0.82, 1.22)
**Lockdown period** ***		
Non-lockdown period	Reference group	Reference group
Lockdown 2	0.95 (0.75, 1.15)	0.93 (0.71, 1.13)
Lockdown 4	1.02 (0.96, 1.11)	1.01 (0.94, 1.10)
Lockdown 5	1.01 (0.91, 1.11)	1.00 (0.90, 1.08)
Lockdown 6	1.03 (0.97, 1.08)	1.00 (0.95, 1.04)
**Christmas period**		
Non-Christmas period	Reference group	Reference group
Christmas 2020	1.05 (0.96, 1.13)	**1.09 (1.01, 1.19)**
Christmas 2021	1.23 (1.15, 1.31)	**1.21 (1.11, 1.31)**

†95% CIs were calculated for this model using a non-parametric bootstrap with 200 samples

*Adjusted for time

**High-risk workers: including aged care workers, hotel quarantine or COVID-19 border control workers, and healthcare workers

***Lockdown 2: 9^th^ July 2020 – 27^th^ October 2020

Lockdown 4: 28^th^ May 2021 – 10^th^ June 2021

Lockdown 5: 16^th^ July 2021 – 27^th^ July 2021

Lockdown 6: 5^th^ August 2021 – 21^st^ October 2021

Lockdown 3 (13^th^ Feb 2021 – 17^th^ Feb 2021) was classified as non-lockdown period in the analysis due to the short period of time

All demographic variables (including Age, Gender, Education, Working environment, Place of residence, Chronic condition and high-risk workers) were measured at baseline.

Of the 688 eligible participants, 595 participants had complete data on the outcome and all covariates and contributed 9,481 surveys to the analysis of heavy episodic drinking. Men had 1.58 times (95% CI: 1.10–2.31) higher odds of reporting heavy episodic drinking compared with women during the study period. Participants aged over 55 (adjusted odds ratio (aOR): 0.29, 95% CI: 0.14–0.60; aOR: 0.32, 95% CI: 0.13–0.81) had lower odds of reporting heavy episodic drinking compared to those aged 18–24 years. Participants with a postgraduate degree had a 0.53 (95% CI: 0.28–0.99) reduced odds of reporting heavy episodic drinking compared to those with a high school or lower degree. Lockdown five (OR: 0.65, 95% CI: 0.48–0.90) and six (OR: 0.76, 95% CI: 0.66–0.86) were strongly associated with a decreased odds of reporting heavy episodic drinking compared to non-lockdown periods. Both the 2020 and 2021 Christmas periods were strongly associated with reporting heavy episodic drinking (Table 4).

**Table 4:** Association between heavy episodic drinking and sociodemographic variables, lockdown period and Christmas period. Results of generalised linear model with GEEs, Optimise, Victoria, Australia, N = 9,481 surveys from 595 participants.

Variable	Heavy episodic drinking	
	Unadjusted Odds Ratio (95% CI)	Adjusted Odds Ratio* (95% CI)
**Age group, years**		
18–24	Reference group	Reference group
25–34	0.73 (0.46, 1.16)	0.82 (0.46, 1.44)
35–44	0.69 (0.40, 1.20)	0.79 (0.41, 1.53)
45–54	0.77 (0.44, 1.32)	0.90 (0.45, 1.78)
55–64	0.25 (0.13, 0.47)	**0.29 (0.14, 0.59)**
65+	0.32 (0.16, 0.65)	**0.32 (0.13, 0.80)**
**Gender**		
Female	Reference group	Reference group
Male	1.90 (1.32, 2.75)	**1.58 (1.09, 2.31)**
**Education**		
High school or lower	Reference group	Reference group
Tertiary education–TAFE/trade certificate	0.96 (0.53, 1.73)	0.96 (0.50, 1.84)
Tertiary education—undergraduate	0.83 (0.52, 1.33)	0.83 (0.49, 1.40)
Tertiary education—postgraduate	0.54 (0.32, 0.91)	**0.53 (0.28, 0.99)**
**Working environment**		
Not working	Reference group	Reference group
Attend workplace	1.59 (0.96, 2.65)	1.04 (0.53, 2.01)
Work from home	1.84 (1.05, 3.22)	1.28 (0.62, 2.61)
Both workplace and working from home	2.14 (1.14, 4.02)	1.62 (0.79, 3.33)
**Place of residence**		
Region	Reference group	Reference group
Metro	1.09 (0.67, 1.78)	0.80 (0.47, 1.37)
**Chronic condition**		
No	Reference group	Reference group
Yes	0.46 (0.30, 0.71)	0.64 (0.40, 1.04)
**High-risk workers** **		
No	Reference group	Reference group
Yes	0.66 (0.41, 1.05)	0.67 (0.39, 1.16)
**Lockdown period** ***		
Non-lockdown period	Reference group	Reference group
Lockdown 2	0.79 (0.50, 1.25)	0.85 (0.51, 1.41)
Lockdown 4	0.75 (0.61, 0.93)	0.81 (0.64, 1.02)
Lockdown 5	0.67 (0.51, 0.89)	**0.65 (0.47, 0.90)**
Lockdown 6	0.74 (0.66, 0.83)	**0.76 (0.67, 0.87)**
**Christmas period**		
Non-Christmas period	Reference group	Reference group
Christmas 2020	1.40 (1.07, 1.82)	**1.37 (1.01, 1.84)**
Christmas 2021	1.37 (1.17, 1.60)	**1.27 (1.06, 1.52)**

* Adjusted for time

** High-risk workers: including aged care workers, hotel quarantine or COVID-19 border control workers, and healthcare workers

*** Lockdown 2: 9th July 2020 – 27th October 2020

Lockdown 4: 28th May 2021 – 10th June 2021

Lockdown 5: 16th July 2021 – 27th July 2021

Lockdown 6: 5th August 2021 – 21st October 2021

Lockdown 3 (13th Feb 2021 – 17th Feb 2021) was classified as non-lockdown period in the analysis due to the short period of time

All demographic variables (including Age, Gender, Education, Working environment, Place of residence, Chronic condition and high-risk workers) were measured at baseline.

## Discussion

Using longitudinal data from 688 participants, this study assessed the frequency of alcohol consumption and heavy episodic drinking over time during the COVID-19 pandemic (October 2020 to August 2022). Our study findings show that the alcohol consumption and heavy episodic drinking among a sample facing increased levels of risk from the pandemic decreased between 2020 and 2022. Consistent with the existing evidence [35, 36], our study found that older adults reported the highest frequency of alcohol consumption, but lower rates of heavy episodic drinking. Men and participants with lower educational attainment were more likely to engage in heavy episodic drinking. Again, this is consistent with both Australian and international research [37–39]. Notably, our study, among people vulnerable to unintended consequences of public health measures used to control COVID-19, suggests that alcohol use did not increase during periods of lockdowns and intense restrictions.

This study found that lockdowns five and six were strongly associated with decreased odds of reporting heavy episodic drinking compared with non-lockdown periods. Notably, lockdown six was the second longest lockdown, at 77 days and the final lockdown imposed. This finding is contrast to some previous studies that found increased alcohol consumption during lockdown periods [40–42], although these studies were from the US and did not focus on specifically vulnerable populations. Previous Australian research focused on the 2020 lockdowns found shifts in drinking locations, but no major changes in levels of consumption [19], while other Australian work suggested that people experiencing specific distresses from the pandemic were more likely to increase drinking [43], but again found limited overall changes in population drinking. No Australian research has examined the specific sub-populations included here or examined the major lockdowns that occurred in 2021 in Victoria, Australia. One potential reason for a decline in heavy episodic drinking, might be the restrictions on public gatherings, along with the shutdown of bars and restaurants, resulting in a decline in heavy drinking occasions [44]. Previous work has also shown that young people’s drinking likely declined in Australia during the pandemic [20], and the reductions in heavy episodic drinking found here likely reflect this shift. Previous international studies suggested that increases in drinking may reflect its use as a negative coping mechanism during the pandemic to manage psychological distress [45, 46], and there is some evidence that severe alcohol-related harms increased in Australia during the pandemic [47], so the reductions identified here may reflect the particular characteristics of our study sample although we expected the sub-populations we recruited to experience higher levels of distress given their increased exposure to risk. On the whole, the negative association between lockdowns and alcohol consumption observed in this sample does alleviate some concern about unintended consequences of public health measures used to control future pandemics, at least where those measures are successful. In contrast to the general finding that Australian alcohol consumption declined, a number of other jurisdictions saw clear increases in population drinking and alcohol harms (e.g. [48–50]). These differences may reflect the additional stress of high levels of exposure to COVID-19 and associated increases in morbidity and mortality compared to the Australian experience, although more detailed cross-national work is needed to better understand the heterogeneity in alcohol trends across the pandemic internationally.

The longitudinal design we used is a strength of this study. By actively following the participants for two years during the COVID-19 pandemic, our study provides new insights on changes in alcohol consumption during the pandemic. Beyond the short-term effects of lockdown onset on drinking behaviours that have been examined in previous studies [19, 20], this study has included five lockdown periods in the analysis, thus offering a more comprehensive view on how extended or recurring lockdowns can impact individuals’ alcohol consumption patterns. Additionally, our study has excellent sample retention, which reduces the potential for selection bias and further increases the internal validity of the results.

Several limitations of this study should be acknowledged. First, our study relied on self-reported frequency of alcohol consumption and heavy episodic drinking, which are subject to recall and other biases. Recall bias and social desirability bias may have led to under- or over-reporting of alcohol consumption therefore the effect of these biases on our study findings is unknown. Secondly, we were only able to assess the frequency of alcohol consumption and heavy episodic drinking by single survey items in this study, as the focus of Optimise was much broader than just substance use. More reliable measurement of alcohol use such as a validated scale may have improved the robustness of our results. In this study, we defined heavy episodic drinking as consuming six or more alcoholic drinks in a single day, which is not consistent with the Australian low risk drinking guidelines of more than four drinks [51], but this item is still broadly reflective of the international literature [52] and trends are unlikely to be affected. It is important to note that survey responses were assigned as being in a category of the lockdown variable based on the date the participant completed the survey. Therefore, a participant may be recalling the past seven days in which not all seven days were “lockdown” or not all seven days were “non-lockdown”. It is important to note though that each participant was on an individual schedule of when they were invited to complete a survey, depending on their day of recruitment. Therefore, it was a small proportion of the sample that would have their alcohol consumption misclassified; misclassification may bias the estimates effects towards the null. Whilst we adjusted the model for important, measured confounders, we did not include an indicator for whether a participant was infected with COVID-19 around the time of survey completion which may have influenced their consumption of alcohol. Lastly, and most importantly, due to the design of the Optimise Study which oversampled high-risk groups, the findings of this study are not broadly generalisable.

This study provides one of the most comprehensive pictures of drinking changes in Victoria during the COVID-19 pandemic by using longitudinal data collected across two years. Although we did not find evidence of an association between frequency of alcohol consumption and lockdown periods in this study, we did find lockdowns five and six were strongly associated with decreased odds of reporting heavy episodic drinking, potentially easing early concerns about the impacts of public health restrictions on alcohol problems in the population.
